# Diet and Gut Microbiota Interaction-Derived Metabolites and Intrahepatic Immune Response in NAFLD Development and Treatment

**DOI:** 10.3390/biomedicines9121893

**Published:** 2021-12-13

**Authors:** Ming Yang, Lea Khoukaz, Xiaoqiang Qi, Eric T. Kimchi, Kevin F. Staveley-O’Carroll, Guangfu Li

**Affiliations:** 1 Department of Surgery, University of Missouri, Columbia, MO 65212, USA; yangmin@health.missouri.edu (M.Y.); lkhoukaz2500@gmail.com (L.K.); qixi@health.missouri.edu (X.Q.); kimchie@health.missouri.edu (E.T.K.); 2 Harry S. Truman Memorial VA Hospital, Columbia, MO 65201, USA; 3 Department of Molecular Microbiology and Immunology, University of Missouri-Columbia, Columbia, MO 65212, USA

**Keywords:** NAFLD, NASH, gut microbiota, metabolite, intrahepatic immunity, clinical trials

## Abstract

Nonalcoholic fatty liver disease (NAFLD) with pathogenesis ranging from nonalcoholic fatty liver (NAFL) to the advanced form of nonalcoholic steatohepatitis (NASH) affects about 25% of the global population. NAFLD is a chronic liver disease associated with obesity, type 2 diabetes, and metabolic syndrome, which is the most increasing factor that causes hepatocellular carcinoma (HCC). Although advanced progress has been made in exploring the pathogenesis of NAFLD and penitential therapeutic targets, no therapeutic agent has been approved by Food and Drug Administration (FDA) in the United States. Gut microbiota-derived components and metabolites play pivotal roles in shaping intrahepatic immunity during the progression of NAFLD or NASH. With the advance of techniques, such as single-cell RNA sequencing (scRNA-seq), each subtype of immune cells in the liver has been studied to explore their roles in the pathogenesis of NAFLD. In addition, new molecules involved in gut microbiota-mediated effects on NAFLD are found. Based on these findings, we first summarized the interaction of diet-gut microbiota-derived metabolites and activation of intrahepatic immunity during NAFLD development and progression. Treatment options by targeting gut microbiota and important molecular signaling pathways are then discussed. Finally, undergoing clinical trials are selected to present the potential application of treatments against NAFLD or NASH.

## 1. Introduction

Nonalcoholic fatty liver disease (NAFLD) is the most common chronic liver disease worldwide, affecting about 25% of the global population [1]. The prevalence of NAFLD is increasing proportionately with the epidemic of obesity and type 2 diabetes (T2D) [2]. Nonalcoholic steatohepatitis (NASH) is the advanced stage of NAFLD with the progression of liver inflammation and cell death with or without liver fibrosis, which can progress to liver cirrhosis and hepatocellular carcinoma (HCC) [3]. In addition, the incidence of NASH is predicted to further increase by up to 56% in the following decade [4]. Although infection of hepatitis C virus (HCV) is the predominant factor causing HCC, NAFLD is the fastest increasing factor that causes HCC in the United States and some European countries [4,5]. With follow-up in seven years, the overall survival rate of patients with NAFLD-HCC was significantly higher than that in patients with HCV and hepatitis B virus (HBV) after treatments [6]. Therefore, a better understanding of the underlying cellular and molecular mechanisms of NAFLD or NASH pathogenesis is helpful to find therapeutic targets to treat NAFLD.

Factors such as lipotoxicity and inflammation can drive NAFLD progression to NASH and ending stage of liver disease [7]. Gut microbiota plays a critical role in the development and progression of NAFLD. Gut microbiota-derived molecules, such as lipopolysaccharides (LPS) and bacterial DNAs, and metabolites such as short-chain fatty acids (SCFAs), can modulate intestinal and systemic immune response [8]. Those gut microbiota-derived metabolites and components can translocate into the liver through the gut-liver axis [9], which are implicated in the initiation and progression of NAFLD. Therefore, finding the key metabolites or components-derived gut microbiota and their function in the pathogenesis of liver disease is helpful for the investigation of NAFLD therapy.

Diet plays an important role in modulating gut microbiota and metabolic pathways in the development of NAFLD [10,11]. Consumption of a high-fat diet (HFD) induces dysbiosis of gut microbiota, leading to metabolic dysfunction, insulin resistance, inflammation, obesity, and T2D [12], a major factor causing NAFLD. In contrast, consumption of a very-low-calorie ketogenic diet (VLCKD) can increase the abundance of SCFA-producing bacteria, such as *Lactobacillus* and *Bifidobacterium* spp., resulting in amelioration of adipose tissue inflammation in obesity and NAFLD [13,14]. In addition, the intervention of VLCKD had a better effect on the reduction in visceral and liver fat accumulation compared to standard low-calorie diet in obese patients [14].

Change of intestinal or hepatic metabolites impacts intrahepatic immune cell profiles, as well as the expression of proinflammatory cytokines and chemokines in the fatty liver. Innate immunity plays an essential role in NAFLD or NASH pathogenesis. For example, the frequency of macrophages was increased in the NASH liver in amylin liver NASH (AMLN) diet-fed mice compared to that in standard chow diet-fed mice [15]. Our research study also showed that monocyte-derived macrophages increased in the liver of wild-type mice fed a choline-deficient, L-amino acid-defined, high-fat diet [16]. Other than that, adaptive immunity, including T cells such as the ratio of T helper (Th) cells/T regulatory cells (Tregs) and B cells, are activated or altered in the development of NAFLD [17,18]. Furthermore, infiltration of inflammatory cells, progression of cell death, and activation of hepatic stellate cells (HSCs) are involved in this process, which may result in NASH progression and liver fibrosis [19]. Many signaling pathways are involved in the proinflammatory response, lipid accumulation, and cell death [3], such as insulin and Wnt signaling pathways. Therefore, targeting the molecules and their associated signaling pathways can potentially treat NAFLD, NASH, and liver fibrosis and prevent NAFLD-related HCC progression.

However, the role of diet and gut microbiota interaction-derived metabolites in modulating intrahepatic immune response remains to be explored. A better understanding of the underlying molecular mechanism is helpful to find a new therapeutic target for NAFLD or potential diagnostic marker. For this purpose, a search was conducted in PubMed, Web of Science, Google Scholar, and Embase with the keywords including NAFLD or NASH, metabolite, gut microbiota, signaling pathway, and immune response in the last five years. The originally retrieved publications were independently reviewed by two authors. The inclusion criteria were (1) the study contained at least three keywords, (2) either animal or human studies. Excluding criteria included (1) studies were abstracts or unpublished studies, (2) studies with similar findings from another study. All the rest studies were carefully reviewed by the authors, and representative findings in the last five years were selected. Few supporting studies prior to this period were added to explain the underlying mechanism.

In this review, we first summarize the latest findings of metabolites that are implicated in the development of NAFLD, as well as the progression to NASH. Then, we investigate the underlying cellular and molecular mechanisms of these metabolites in hepatic immunity in animal models to study NAFLD or NASH or clinical samples. Finally, we summarize the currently ongoing clinical trials to evaluate potential therapeutic reagents by targeting key molecules or proteins for NAFLD and NASH treatment.

## 2. Gut Microbiota-Derived Metabolites in the Pathogenesis NAFLD and NASH

LPS, a major component of Gram-negative bacterial cell membrane, plays a pivotal in the pathogenesis of mouse and human NAFLD via Toll-like receptor 4 (TLR4) signaling pathway [20]. In addition to gut microbial components, metabolites derived from gut microbiota also impact hepatic function, including amino acids, secondary bile acids, ethanol, lipids, and SCFAs. For example, a tryptophan-derived metabolite indole-3-propionic acid (IPA) by gut microbiota showed anti-NASH ability in rats by reducing gut LPS leakage, which can activate hepatic macrophages to produce proinflammatory cytokines (e.g., tumor necrosis factor (TNF)-α and interleukin (IL)-1β) to cause liver inflammation and fibrosis [21,22]. An updated summary in the following context is to describe the function of metabolites in the development of NAFLD from recent research findings.

### 2.1. Amino Acids

Plasma amino acids (AAs), such as glutamate and valine, are shown to increase in NAFLD patients with or without obesity compared to non-NAFLD controls [23]. Hoyles et al. reported that dysregulation of branched-chain amino acid and aromatic amino acid metabolism was positively associated with hepatic inflammation and steatosis in non-diabetic obese women, resulting from gut microbial dysbiosis with the richness of genes for dietary lipid metabolism and LPS biosynthesis [24]. This study also showed that phenylacetic acid (PAA), a microbiota-derived metabolite from aromatic amino acid phenylalanine, was positively associated with hepatic steatosis. Another study showed that limiting glycine source or inhibiting glycine biosynthetic genes such as alanine-glyoxylate aminotransferase 1 (AGXT1) accelerated diet-induced NASH and hyperlipidemia [25]. Treatment with a tripeptide DT-109 (Gly-Gly-L-Leu) ameliorated mouse NASH features induced by a high-fat, cholesterol, and fructose diet by enhancing liver mitochondrial fatty acid β-oxidation (FAO) and stimulating de novo glutathione synthesis [25]. Thus, modulating AA metabolites can potentially inhibit the progression of NAFLD.

### 2.2. Bile Acids

Bile acids (BAs) play important roles in NAFLD pathogenesis by modulating hepatic lipid and glucose metabolism, consisting of primary and secondary BAs [26]. Primary BAs such as chenodeoxycholic acid (CDCA) are produced in the liver, while gut microbiota can metabolize them to secondary BAs such as deoxycholic acid (DCA) [9]. BA receptors such as nuclear Farnesoid X receptor (FXR) and the Takeda G protein-coupled receptor 5 (TGR5) are important molecules that are involved in the modulation of energy metabolism and inflammation during metabolic disorders, including NAFLD [27]. For example, a high-fat diet (HFD)-induced development of NAFLD has been reported to be associated with a decrease in the ratio of non-12α-OH BAs (e.g., HDCA/Hyodeoxycholic)/12α-OH BAs (e.g., DCA) with downregulation of FXR and TGR5 and upregulation of cytochrome P450 family 7 subfamily A member 1 (CYP7A1) and TLR4 [28]. Modulating gut microbiota with an antibiotic cocktail can alleviate HFD-induced hepatic steatosis and inflammation in hamsters via upregulating cytochrome P450 family 7 subfamily B member 1 (CYP7B1) to increase hydrophilic BA synthesis [29].

### 2.3. Choline Metabolism

Choline can be metabolized by the gut microbiota to trimethylamine (TMA), which is absorbed in the liver and further converted to trimethylamine N-oxide (TMAO) by flavin-containing monooxygenase 3 (FMO3) [30]. In addition to choline, TMA precursors such as L-carnitine and betaine are rich in diets (e.g., red meat and eggs), and overconsumption of these diets can increase TMAO in plasma to promote NAFLD through activation of oxidative stress, unfolded protein response, and change of bile acid metabolism [31]. A prospective study showed that plasma levels of TMAO were positively associated with all-cause mortality in human NAFLD patients but not in non-NAFLD patients, which was independent of traditional risk factors, such as triglyceride glucose, and body mass index (BMI) [32]. TMA-producing bacteria consist of enzymes choline-TMA lyase (CutC), carnitine oxygenase (CntA), and betaine reductase (GrdH), such as *Firmicutes* [32,33,34,35]. In addition, several choline-deficient diets were applied to induced mouse NASH and liver fibrosis models [36].

### 2.4. Ethanol

Excessive consumption of alcohol causes alcohol fatty liver disease (AFLD). Endogenous ethanol produced by gut microbiota can impair mitochondrial function and promotes NAFLD development [37]. Gavage of ethanol-producing gut microbiota (e.g., *Klebsiella pneumoniae*) to mice can increase ethanol production, increase liver injury, and impair mitochondrial function in mice, indicating a causative factor for NAFLD [37]. Fasting ethanol concentration in plasma has been shown to be positively associated with insulin resistance in children with NAFLD compared to controls [38]. Further studies in mice also showed that impaired activity of alcohol dehydrogenase (ADH) in the liver tissue is the major cause of ethanol concentration increase instead of an increase in endogenous ethanol synthesis [38]. Thus, ethanol either produced endogenously by gut microbiota or caused by impaired ADH in the liver can impact NAFLD progression.

### 2.5. Fiber

Dietary fibers (DF) consist of carbohydrate polymers resistant to digestive enzymes in the small intestine, which can be digested by bacteria in the large intestine [39]. DF can be divided into soluble and insoluble forms based on the solubility in water, and soluble fibers can be degraded into SCFAs [40]. Supplementation of oligofructose, a DF, is helpful to reduce body weight in obese adults [41]. Obese patients with consumption of higher insoluble fiber consumption (≥7.5 g/day) had improvement in the fatty liver index, hepatic steatosis index, and NAFLD liver fat score, while patients with fruit fiber consumption (≥8.8 g/day) showed significant improvements in gamma-glutamyl transferase (GGT), alanine aminotransferase (ALT), and aspartate aminotransferase (AST) [42]. A clinical trial study also showed that consumption of a low-carbohydrate and high-fiber diet with education can effectively reduce the body weight and body fat of NAFLD patients and improve metabolic disorders [43]. One of the underlying mechanisms is to change gut permeability, as evidenced by the reduction in serum levels of zonulin in NAFLD patients with DF [44].

Fermentation of DF can impact the diversity of gut microbiota. For example, a meta-analysis revealed that DF intervention can increase the abundance of *Bifidobacterium* and *Lactobacillus* genera compared to placebo or low-fiber consumption, which is associated with a high concentration of butyrate in feces [45]. Consumption of brans such as oat and rye containing 50% DF can reduce body weight gain and ameliorate Western diet (WD)-induced liver inflammation via altering gut metabolism such as indole production [46].

### 2.6. Short-Chain Fatty Acids

SCFAs, consisting of acetate, propionate, and butyrate, are produced by gut microbiota from dietary fibers and starch. They play important roles in energy metabolism, tissue homeostasis, and immune regulation. Here, we discuss their roles in the pathogenesis of NAFLD.

#### 2.6.1. Acetate

Oral administration of branched-chain amino acids (BCAAs), including leucine, isoleucine, and valine, significantly increased the abundance of gut *Ruminococcus flavefaciens* and portal acetic acid concentration, resulting in a reduction in hepatic fat accumulation [47]. In addition, a molecular mechanism study showed that BCAA treatment inhibited the expression of lipogenesis-related enzymes such as fatty acid synthase (FAS) and acetyl-CoA carboxylase (ACC). It has been reported that both butyrate and propionate show predominantly anti-obesity effects, whereas acetate has more potential to promote obesity and lipogenesis in the liver and adipose tissue [48].

#### 2.6.2. Propionate

A randomized controlled trial study showed that dietary supplementation with inulin that is mainly metabolized into acetate in the colon increased intrahepatocellular lipid. In contrast, dietary supplementation of inulin-propionate ester, which is designed to deliver propionate to the colon and to attenuate the acetate-mediated increase in intrahepatocellular lipid [49].

#### 2.6.3. Butyrate

Supplementation with grape polyphenols reduced Western diet (WD)-induced adiposity and hepatic steatosis in mice by increasing the abundance of *Akkermansia muciniphila* and butyrate and sugar expenditure in the distal intestine [50].

**Figure 1:**
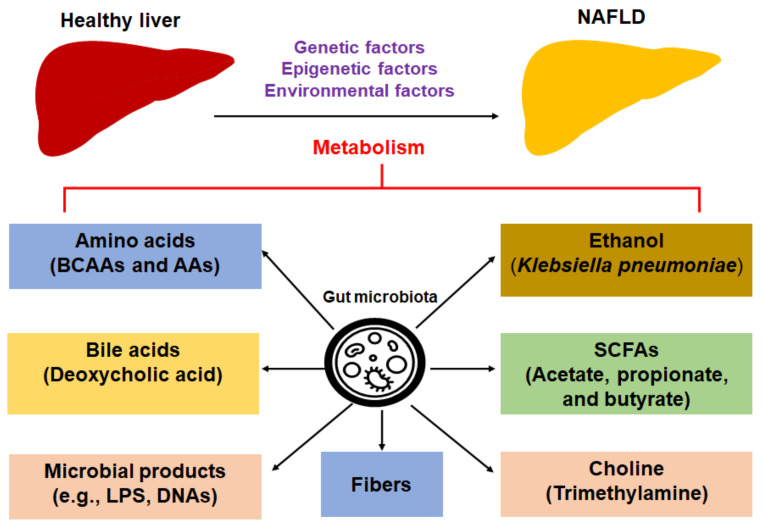
Dietary metabolites or metabolites derived from gut microbiota impact the progression of NAFLD. Abbreviations: AAA, aromatic amino acid; BCAA: branched-chain amino acid; LPS, lipopolysaccharide; NAFLD, nonalcoholic fatty liver disease; SCFAs, short-chain fatty acids.

Overall, the dietary metabolites or metabolites derived from gut microbiota impact the progression of NAFLD and NASH (Figure 1).

## 3. Intrahepatic Immunity in NAFLD and NASH in Diet-Induced Murine Models and Human Patients

The intrahepatic immune response plays an essential role in the progression of NAFLD/NASH. Gut microbiota-derived metabolites and components circulating in the portal vein system can enter the liver to modulate intrahepatic immunity to impact NAFLD. This process is involved in a complicated communication among different liver non-parenchymal cells, including macrophages, monocytes, T cells, B cells, neutrophils, and HSCs [51]. Herein, we update some recent findings in this field to explore new molecules or cell subtypes in the pathogenesis of NALFD. Animal models of steatosis, NAFLD, and NASH have been summarized in recent publications [52], which are briefly mentioned with the discussion of immune activation.

### 3.1. Macrophages/Monocytes

The composition of liver macrophages was altered in mice fed a high-fat high-sucrose diet (60% fat and 10% sucrose), with a decrease in liver resident macrophage Kupffer cells (KCs) and an increase in monocyte-derived macrophages (MdMs) detected by single-cell RNA sequencing (scRNA-seq) [53]. A subset of MdMs shows the phenotype of lipid-associated macrophages (LAMs) characterized by the expression of triggering receptor expressed on myeloid cells 2 (Trem2), cluster of differentiation (CD)63, CD9, and glycoprotein nonmetastatic melanoma protein B (Gpmnb) [53]. In addition, Cc chemokine receptor (CCR)2 expression is critically important for the recruitment of this population. Gut microbiota-derived tryptophan metabolites tryptamine and indole-3-acetate (I3A) can attenuate the expression of TNF-α, IL-1β, and MCP-1 on macrophages exposed to palmitate and LPS [54]. Those cytokines expressed by macrophages can promote NAFLD progression.

### 3.2. NK Cells

The number of natural killer (NK) cells was increased in a methionine- and choline-deficient diet (MCD)-induced mouse NASH liver via C-X-C motif chemokine ligand (CXCL)10/chemokine receptor (CXCR)3 signaling [55]. These intrahepatic NK cells expressed low levels of protein Ki67, indicating a reduced proliferation ability. In addition, depletion of NK cells induced hepatic infiltration of MdMs with M2-like phenotype, advancing liver inflammation and fibrosis [55]. Another study showed that CD56^bright^NK cells decreased in intrahepatic lymphocytes in NAFLD patients, while CD56^dim^NK cells increased compared to that in healthy controls, indicating the complex roles of each subtype of NK cells in NAFLD [56]. However, another study showed that there was only a minor change in NK cell activation and inhibitory markers from NASH patients, except natural killer group 2 member D (NKG2D) [57]. Natural cytotoxicity triggering receptor 1 (NKp46)^+^ NK cells can inhibit the progression of NASH and liver fibrosis via suppressing the expression of profibrogenic genes as well as M2 polarization (anti-inflammatory phenotype) of liver macrophages [58]. Therefore, the role of NK cells is dependent on their subtypes.

### 3.3. NKT Cells

Activation of invariant natural killer T (iNKT) cell subsets was shown in choline-deficient L-amino acid-defined HFD (CDAHFD)-induced murine NASH, accompanying the accumulation of plasmacytoid dendritic cells (pDCs) [59]. In addition, the frequency of iNKT cells was increased in peripheral blood mononuclear cells (PBMCs) from NASH patients compared to that in healthy controls. The axis of CXCR6/CXCL16 plays an essential role in the recruitment of NKT cells in fatty liver, liver fibrosis, and liver cancer [60,61]. Gut microbiota such as *Clostridium* spp. induced secondary bile species (sBAs) activated liver sinusoidal endothelial cells (LSECs) to produce the chemokine CXCL16 to attract accumulation of hepatic CXCR6^+^NKT cells [62]. CD1d-deficient mice lacking NKT cells on a high-fat high carbohydrate (HFHC) showed reduced body weight and hepatic triglyceride content, mRNA expression of α-smooth muscle actin (α-SMA), collagen type 1 alpha 1 (Col1α1) and alpha 2 (Col1α2), and infiltration of macrophages, with improved NAFLD activity scores [63]. Overall, NKT cells are normally increased in the liver, accompanying the development of NAFLD and NASH.

### 3.4. Neutrophils

Neutrophils are one of the first response cells that are recruited to the injury site to participate in the inflammatory response and tissue repair. Neutrophil depletion treated with antibody 1A8 (200 μg/mouse per week for four times) can reduce body weight gain and attenuate liver lipid accumulation with activation of lipid β-oxidation in HFD-fed mice compared to mice treated with isotype control [64]. Neutrophil depletion was also associated with a reduction in expression of inflammatory cytokines, such as TNF-α, IL-6, and monocyte chemoattractant protein-1 (MCP-1/CCL2) [64].

### 3.5. CD4 T Cells

Different subtypes of CD4^+^ T cells play different roles in NAFLD pathogenesis. Fatty acid composition (e.g., the ratio of C16:1n7/C16:0) can modulate the frequency of CD4^+^ T cell profiles in PBMCs of NAFLD patients, with an increase in CD25^+^CD45^+^CD4^+^ T cells and a decrease in PD1^+^CD4^+^ T cells [65].

Obesity increased the accumulation of inflammatory hepatic CXCR3^+^ T helper 17 (Th17) cells and concomitant expression of IL-17a, interferon (IFN)-γ, and TNF-α, resulting in NAFLD progression [66]. Cellular metabolism impacts the inflammatory phenotype of hepatic Th17 cells, especially by pyruvate kinase M2 (PKM2)-mediated glycolytic pathway [66]. The ratio of Th17 and regulatory T (Treg) cells is critically important in the pathogenesis of NAFLD and liver inflammation. Feeding an HFD increased the frequency of liver Th17 cells; meanwhile, it caused a decrease in Tregs in mice compared to ND feeding mice, resulting in an increased Th17/Treg ratio, progression of NAFLD, and liver inflammation [67]. IL-17^+^CD4^+^ T cells were significantly increased in the liver during NAFL to NASH progression [68]. The increase in Th17 cells in NASH patients was positively correlated with an increased blood concentration of LPS [69].

Hepatic infiltration of Tregs was increased in CD62L-deficient mice, which was associated with less hepatic lipid accumulation, reduced liver fibrosis, and improved insulin resistance [70]. However, adoptive transfer of Tregs from healthy wild-type mice to mice fed a high-fat, high-fructose diet (HFHFD) promoted hepatic steatosis due to infiltration of Tregs in subcutaneous adipose tissue and/or a decrease in Th1 cells [71].

### 3.6. CD8 T Cells

Liver CD8^+^ T cells were increased in obese patients with NASH, which was associated with the expression of α-SMA, a marker of HSC activation [72]. Depletion of liver CD8^+^ T cells reduced hepatic macrophages and α-SMA expression in obesity or hyperlipidemia-induced NASH mice, but not in lean mice [72]. RNA-seq data showed that perforin deficiency increased proinflammatory cytokine expression in hepatic CD8^+^ T cells in mice with NASH [73]. Perforin-deficient mice fed with a methionine- and choline-deficient diet (MCD) displayed an increase in CD8^+^ T cell accumulation and activation with the expression of proinflammatory cytokines, but not CD4^+^ T cells and NK cells. Ex vivo studies revealed that microbiota-derived extracts in NAFLD-HCC patients compared to that can induce an immunosuppressive phenotype in human PBMCs, characterized by a suppression of CD8^+^ T cells and expansion of Tregs [19]. NAFLD promotes CD8^+^ T cell activation and suppresses its cytotoxicity to tumor cells by inducing immune tolerance.

### 3.7. B Cells

Fecal microbiota transplantation (FMT) of gut microbiota from human NAFLD patients into recipient mice can accelerate NASH progression via inducing accumulation and activation of liver B cells [74]. ScRNA-seq data showed that intrahepatic B cells in NASH mice display proinflammatory phenotype with activation of myeloid differentiation primary response protein 88 (MyD88) signaling pathway [74]. Furthermore, depletion of B cells suppressed NASH progression, whereas adoptive transfer of B cells from NASH liver can induce NASH, indicating the pathogenic role of B cells in NASH.

**Figure 2:**
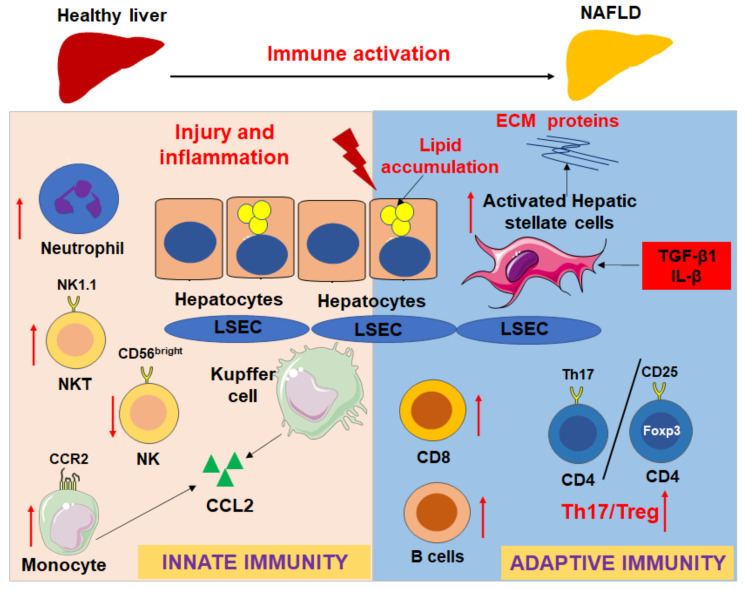
Innate and adaptive immune responses in the progression of NAFLD and liver fibrosis. Red arrows show that the immune cells will be recruited into the fatty liver during NAFLD development, such as CCR2^+^ monocytes/macrophages and neutrophils; the ratio of Th17/Tregs increases, NKT cell, CD8 T cells, and B cells are activated and increased in different extend according to different models; however, CD56^bright^NK cells are decreased. The immune activation and hepatocyte injury will impact the activation of hepatic stellate cells (HSCs) to express extracellular matrix (ECM) proteins via upregulation of profibrotic and proinflammatory cytokines, such as TGF-β1 and IL-β.

Activation of HSCs, the major cells that contribute to liver fibrosis, is mediated by the activation of intrahepatic immunity during NASH. For example, proinflammatory cytokines such as TNF-α, transforming growth factor (TGF)-β1, and IL-1β expressed by intrahepatic macrophages can activate HSCs to promote the progression of liver fibrosis and NASH [16]. In contrast, a recent study showed that tissue-resident memory CD8^+^ T cells can trigger apoptosis of activated HSCs via Fas (TNF receptor superfamily, member 6)/FasL-mediated signaling [75]. Therefore, the immune activation, hepatocyte injury, and activation of HSCs are cross-talked with each other during NAFLD development and progression (Figure 2).

## 4. Molecules Involved in the Recruitment of Immune Cells in NAFLD and NASH

The recruitment of immune cells into the fatty liver plays a critical role in the pathogenesis of NAFLD/NASH. Chemokines and their receptors are the key factors involved in the recruiting process. For example, CCL2/CCR2 and CXCL9/10/CXCR3 signaling pathways are involved in the migration of myeloid cells and T cells [66,76,77,78]. Another study showed that gut-derived lymphocytes from mesenteric lymph nodes (MLN) can migrate to the liver via CCL5 signaling and induce liver T cell activation and injury [79]. In this review, we discuss some recently explored molecules that are associated with the infiltration of immune cells during NAFLD development and progression.

### 4.1. Integrins

Hepatic accumulation of integrins α4β7^+^CD4^+^ T cells was positively associated with hepatic steatosis, inflammation, and fibrosis via its ligand mucosal addressin cell adhesion molecule 1 in a Western diet (WD)-fed mice [80]. Another study showed that β7-Integrin-deficient mice exhibited more inflammatory cell infiltration in the livers of mice fed with HFD, especially neutrophils, promoting NASH progression [81].

### 4.2. Selectin

A soluble form L-selectin/CD62L was dramatically increased in the liver in patients with NASH. CD62L-deficient mice showed dampened NASH features compared to wild-type mice, including less hepatic lipid accumulation, reduced liver fibrosis, and improved insulin resistance [70]. Hepatic infiltration of Tregs was increased in CD62L-deficient mice. Similarly, treatment with anti-CD62L antibody protected HFD-induced NASH in mice [70].

### 4.3. Runt-Related Transcription Factor 2 (Runx2)

Infiltration of hepatic infiltration of macrophages in HFD-induced NAFLD mice was associated with an increase in hepatic Runx2 expression [82]. Both in vivo and in vitro studies further revealed that the expression of receptor activator of nuclear factor-κB (NF-κB) ligand (RANKL) was positively correlated with Runx2 expression [82]. Runx2 was shown to be more specifically expressed in activated HSCs in NAFLD mice, which can modulate the expression of monocyte chemotactic protein 1 (MCP-1) to increase liver infiltration of macrophages [83].

## 5. Treatment Options for NAFLD and NASH Based on Modulation of Gut Microbiota, Intrahepatic Immunity, and Metabolic Signaling Pathways

Many treatment agents have been tested in preclinical animal studies for the treatment of NAFLD or NASH with promising effects, including modulation of gut microbiota, FXR modulators, targeting chemokines and their receptors, anti-inflammatory or antioxidant agents, and modulation of fibroblast growth factors (FGFs) and microRNAs (miRNAs).

### 5.1. Modulation of Gut Micorbiota

#### 5.1.1. Bariatric Surgery (BS)

A prospective cohort in Japan showed that the prevalence of NAFLD and NASH was 82.4% and 77.5%, respectively, in morbidly obese patients [84]. BS treatment in morbidly obese patients with NASH resulted in 85% of the disappearance of NASH and reduction in histological features, including steatosis, hepatocellular ballooning, and lobular inflammation [85]. A 5-year follow-up of NASH patients with BS showed that NASH was resolved in 84% of patients, and fibrosis was ameliorated in 70.2% of patients. In addition, no significant recurrence was shown in patients with resolution of NASH in 5 years [86].

#### 5.1.2. Fecal Microbiota Transplantation

FMT has been tested as a therapeutic strategy to prevent and treat different diseases associated with gut microbiota dysbiosis. FMT is a procedure to transfer healthy donor stool into the gastrointestinal tract of the patient in order to restore the balance of gut microbiota. For example, FMT is an effective and safe treatment for the recurrence and reduction in severe *Clostridium difficile* infection (CDI) induced by gut dysbiosis [87,88]. The serum level of proinflammatory cytokines (e.g., TNF-α and IL-1β) was significantly reduced in CDI patients with FMT [87], the inducing factor for NAFLD. Eight-week FMT improved gut microbiota dysbiosis with increased abundances of the beneficial bacteria *Christensenellaceae* and *Lactobacillus* and intestinal tight junction protein ZO-1, and reduced hepatic lipid accumulation, proinflammatory cytokines, and NAFLD activity score (NAS) in HFD-fed mice [89]. In addition, hepatic expression of IFN-γ and IL-17 was decreased post FMT. A clinical study showed that FMT in NAFLD patients did not improve insulin resistance and hepatic proton density fat fraction but improved the intact of small intestinal barrier [90]. Still, more clinical trials are expected to further validate the efficacy of FMT in NAFLD/NASH patients.

#### 5.1.3. Probiotics

Treatment with probiotics significantly ameliorated HFD-induced NAFLD in rats by decreasing the abundance of pathogenic bacteria and upregulating the bile acid receptor FXR/FGF15 signaling pathway [91]. A meta-analysis showed that probiotics/synbiotics were helpful to reduce hepatic steatosis, inflammation, liver stiffness measured by elastography in patients with NAFLD [92]. In addition, treatment with probiotics but not synbiotics was associated with a reduction in body mass index. Pediatric NAFLD patients treated with a probiotic capsule, including *Lactobacillus acidophilus* (ATCC B3208), *Bifidobacterium lactis* (DSMZ 32269), *Bifidobacterium bifidum* (ATCC SD6576), *Lactobacillus rhamnosus* (DSMZ 21690) for 12 weeks showed reduced liver injury and a higher percentage of normal liver sonography in compared to placebo treatment [93]. Administration of *Bacteroides uniformis* (CBA7346), a strain isolated from the healthy human gut, can ameliorate liver injury, inflammation, and lipid accumulation in NAFLD mice induced by feeding an HFD via improving insulin resistance and regulating de novo lipogenesis-related proteins, such as fatty acid synthase (FAS) and peroxisome proliferator-activated receptor-gamma (PPARγ) [94].

### 5.2. FXR Modulators

Clifford et al. showed that FXR activation both in mice and humans can specifically decrease the levels of monounsaturated fatty acids (MUFA) and polyunsaturated fatty acids (PUFA) in the liver [95]. FXR agonist GSK2324 suppressed hepatic lipid accumulation via suppressing lipogenesis in the liver and lipid absorption in the intestine [95]. Treatment with FXR agonist cilofexor reduced portal pressure and hepatic hydroxyproline product, as well as the expression of Col1a1, platelet-derived growth factor receptor beta (PDGFR-β), and desmin in NASH rats [96], indicating amelioration of liver fibrosis.

### 5.3. Targeting Chemokines/Chemokine Receptors

Chemokines and their receptors, such as CCL25 and CCR9, play important roles in the hepatic infiltration of macrophages and other immune cells in NAFLD/NASH [97,98]. Therefore, inhibiting this axis may prevent liver inflammation and liver fibrosis. Treatment with CCR9 antagonist CCX282-B (vercirnon) inhibited fibrosis progression in mice with NASH [98]. Blocking CCL24 with a monoclonal antibody significantly reduced liver fibrosis and inflammation in methionine choline-deficient (MCD) and STAM (streptozotocin + HFD) mouse models and in thioacetamide (TAA)-treated rat model [99].

### 5.4. Modulation of FGFs

Treatment with aldafermin, an engineered analog of FGF19, markedly reduced serum BAs, specifically hydrophobic BAs, such as DCA, lithocholic acid (LCA), glycodeoxycholic acid (GDCA), glycochenodeoxycholic acid (GCDCA), and glycocholic acid (GCA) in NASH patients [100]. In addition to prebiotics, natural medicine such as the traditional Chinese medicine Salvia-Nelumbinis naturalis can activate intestinal FXR-FGF15 signaling to decrease hepatic CD68^+^ macrophages and expression of inflammatory cytokines IL-1β and TNF-α [101].

### 5.5. Anti-Inflammatory and Anti-Oxidative Agents

Natural polyphenols such as resveratrol with anti-inflammatory and antioxidant properties show potential efficiency against NAFLD [102]. Polyphenol showed multiple effects, including reduction in body weight gain and hepatic fat accumulation, improvement of insulin resistance, and amelioration of oxidative stress, mitochondrial dysfunction, and ER stress [103]. In addition, they can decrease both serum and liver proinflammatory cytokines that contribute to the fatty liver [104]. Treatment with methyl brevifolincarboxylate, a natural polyphenolic compound, reduced lipid metabolism and inflammatory markers, such as TNF-α, IL-6, and IL-1β, via modulating 5′ adenosine monophosphate-activated protein kinase (AMPK)/NF-κB signaling pathway [105]. However, clinical studies are still needed to confirm the function of polyphenols.

Administration of hydro-alcoholic extract of spinach reduced the expression of proinflammatory cytokine TNF-α and enhanced the expression of PPAR-γ in the livers of NAFLD rats at prevention and treatment phases [106]. Dietary vitamin E such as α-tocopherol has potential protective effects against steatosis [107].

### 5.6. miRNAs

MicroRNAs (miRNAs) play important roles in regulating cell apoptosis, migration, and lipid metabolism during the development of NAFLD [108], which may function as diagnosis markers (e.g., miR-144-3p and miR-200b-3p) [109]. Keeping the balance of Th17/Treg ratio via modulating miR-195 expression can inhibit CD40 expression to ameliorate NAFLD in rats. In contrast, anti-miR-195 treatment aggregated NAFLD by interrupting Th17/Treg balance [110]. Upon a high-fat, high-cholesterol, high-sugar diet feeding, miR-155 KO mice displayed less liver injury, decreased steatosis, and attenuation in fibrosis compared to wild-type mice [111].

**Figure 3:**
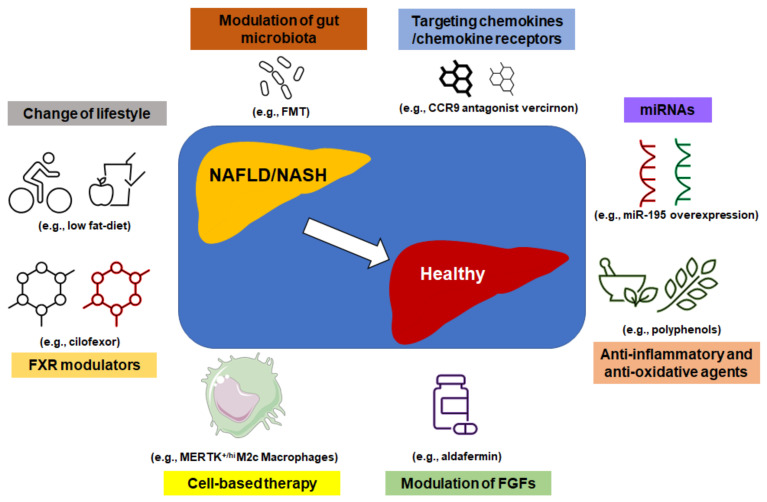
Treatment options for NAFLD. There are several options to accelerate the reverse of NAFLD even NASH, including modulation of gut microbiota, targeting chemokine/chemokine receptor signaling, change of lifestyle, modulation of miRNAs, Farnesoid X receptor (FXR), and fibroblast growth factors (FGFs), cell-based therapy, anti-inflammatory, and anti-oxidative agents, as well as others.

The above-discussed treatment options are summarized in a figure (Figure 3). In addition, cell-based therapy by adoptive transfer cells to NAFLD mice shows a therapeutic effect. For example, injection of anti-inflammatory MER receptor tyrosine kinase (MERTK)^+/hi^M2c-macrophages to NAFLD mice increased serum level of high-density lipoprotein (HDL) and decreased total NAFLD pathological score via reducing liver inflammation, cell death, and fibrosis [112].

## 6. Clinical Trials for NAFLD Treatment

Many treatment agents have been tested in clinical trials for the treatment of NAFLD or NASH with promising effects, including chemokine receptor antagonist (e.g., cenicriviroc, a dual antagonist of CCR2 and CCR5), FXR agonist (e.g., obeticholic acid), modulation of FGF (e.g., aldafermin, an analog of FGF19), PPAR agonist (e.g., lanifibranor, a pan-PPAR agonist), diet intervention (e.g., low-calorie diet), anti-inflammatory or antioxidant agents (e.g., omega-3), and modulation of gut microbiota (e.g., synbiotics). Representative trials were selected with clinical results in the last 5 years before 11 October 2021 (Table 1).

## 7. Conclusions

NAFLD is the most chronic liver disease in the global population, and its incidence increases with the prevalence of obesity and T2D. Currently, NAFLD is the most increasing factor to induce primary liver cancer, HCC. However, there are no currently available FDA-approved treatments for NAFLD. Gut microbiota-derived metabolites and components play pivotal roles in the development and progression of NAFLD. Preclinical studies and clinical trials have been processed to evaluate potential treatment options for NAFLD and NASH, including synbiotics, omega-3, CCR2/5 antagonists, FXR agonists, and so on. A combined treatment such as combined medical treatment and physical activity could reduce the treatment time and improve the outcome. Although preclinical animal studies show the effects of pre-/probiotics and FMT, more clinical trials are waiting to verify the efficacy of balancing gut microbiota profile in patients with NALFD/NASH. In the future, meta-omics, including metabolomics with bioinformatic analysis, should be applied to search for early diagnostic markers and therapeutic targets for NAFLD and NASH.

## Figures and Tables

**Table 1:** Current clinical trials for NAFLD and NASH treatment.

Trial Number	Phase	Agents	Results	References
NCT02217475	2	Cenicriviroc	Treatment with cenicriviroc (CVC), a dual antagonist of CCR2 and CCR5, ameliorated liver fibrosis but did not worse steatosis compared to placebo treatment.	[36]
NCT03008070	2	Lanifibranor	Treatment with lanifibranor, a pan-PPAR agonist, decreased liver enzyme levels and inhibited lipid accumulation, inflammation, and fibrosis.	[113]
IRCT2016102530489N1	2–3	Omega-3	Supplementation with omega-3 for 12 weeks with 2000 mg per day can improve fatty liver and visceral adiposity indexes.	[114]
NCT02443116	2	Aldafermin	Treatment with aldafermin (1 mg) daily for 24 weeks, an analog of FGF19, significantly reduced liver fat content and improved liver injury, and improved liver fibrosis in a higher percentage of NASH patients, compared to placebo.	[115]
NCT02912260	2	Resmetirom	Treatment with resmetirom, a liver-directed, orally active, selective thyroid hormone receptor-β agonist, significantly reduced liver fat accumulation after 12 weeks or 36 weeks in patients with NASH.	[116]
NCT01265498	2	Obeticholic acid	Treatment with obeticholic acid (OCA), a farnesoid X receptor agonist, increased total low-density lipoprotein (LDL) particle concentration and reduced a reduction in total high-density lipoprotein (HDL) particle concentration at 12 weeks.	[117]
NCT01680640	2	Synbiotic	Administration of a synbiotic combination of probiotic and prebiotic agents for a year changed fecal microbiome but did not ameliorate fatty liver and liver fibrosis.	[118]
NCT04038853	4	Vitamin D	Over twelve-month treatment of low-medium dose supplementation of vitamin D (1000 IU/day) decreased transient elastography (FibroScan) indices of liver steatosis and fibrosis (liver stiffness measurement) in adult NAFLD patients.	[119]
NCT02679417	None	Exercise and dietary change	Both moderate-intensity aerobic training and resistance training with dietary modification can effectively reduce liver fat and improve insulin resistance in NAFLD patients.	[120]
IRCT20100524004010N23	None	*Bacillus coagulans* plus inulin	Twelve-week supplementation with *Bacillus coagulans* plus inulin is beneficial for the treatment of NAFLD and its related inflammation without any significant effects on related cardiovascular risk factors.	[121]
ISRCTN85177264	None	A very-low-calorie diet	With a very low-calorie diet (VLCD) intervention for a maximum of 12 weeks, 34% and 68% of patients achieved and sustained ≥10% and ≥5% weight loss at 9-month follow-up, respectively. For NAFLD patients who completed the dietary intervention, VLCD can improve liver health, cardiovascular risk, and metabolic health in those completing the intervention.	[122]

## Data Availability

All the data supporting reported results can be found in this manuscript.
